# Workplace Adversity Among Critical Care Nurses: Associations With Key Organisational Factors

**DOI:** 10.1111/nicc.70399

**Published:** 2026-02-15

**Authors:** Candice Bowers, Dalena Van Rooyen, David Morton

**Affiliations:** ^1^ Department of Nursing Science Nelson Mandela University Gqeberha South Africa

**Keywords:** critical care nurses, intensive care unit, job demands‐resources model, organisational factors, workplace adversity

## Abstract

**Background:**

Workplace adversity in intensive care units (ICUs) is a multifaceted phenomenon resulting from a combination of organisational, discipline‐specific and external influences. Guided by the Job Demands‐Resources (JD‐R) model, this study explored the organisational factors associated with workplace adversity among critical care nurses in ICUs. Currently, there is a paucity of research that explicitly analyses workplace adversity as a central concept.

**Aim:**

To determine organisational factors associated with workplace adversity from the perspective of critical care nurses working in adult, paediatric and neonatal ICUs in public and private hospitals in the Eastern Cape Province, South Africa.

**Study Design:**

The study employed a quantitative, cross‐sectional correlational survey design. The target population consisted of critical care nurses. A census sampling method was employed, and data were collected using a structured self‐report questionnaire. Descriptive and inferential statistics were used to conduct the data analyses using Statistica Version 13.5.

**Results:**

Three hundred and fifty‐three (353) questionnaires were completed. Respondents highlighted the following organisational factors as the primary contributors to workplace adversity: 90% (*n* = 312) staff shortages, 70% (*n* = 243) insufficient nurse‐to‐critically ill patient ratios, 57% (*n* = 197) limited access to professional development and 50% (*n* = 171) unsuitable leadership style of direct line managers.

**Conclusions:**

Unaddressed organisational factors causing workplace adversity have the potential to destabilise ICU environments, undermine staff well‐being, patient care and overall healthcare system performance. Identifying and mitigating these factors is, therefore, essential to sustaining effective and resilient ICU settings.

**Relevance to Clinical Practice:**

Direct line managers working in ICUs should proactively identify and address the organisational factors that contribute to workplace adversity in their respective units.

## Impact Statements

- What is known about the topic
  ○ Recent qualitative studies directly address workplace adversity as a construct, exploring factors such as resilience, stress and burnout as causes.
- What this paper adds
  ○ Few quantitative studies have addressed the concept of organisational factors associated with workplace adversity among critical care nurses.
  ○ Additionally, this study provided unique insights specifically into demographic variables and variances among hospital groups.

## 1 Introduction and Background

Workplace adversity has been defined as challenges affecting a single staff member, a team, or an entire institution [1]. Such challenges include the trials employees face, resulting in a decline in physical health, emotional health or feelings of stress [2]. The sources of workplace adversity vary across different organisations [1], and these emanate from within working teams, managerial structures, or even the nature and culture of institutions [3]. However, studies show that interventions that attempt to address workplace adversity are often focused at the level of the individual but appear to ignore the organisational factors, such managerial inadequacies [4, 5]. South African studies reveal a range of challenges in ICUs but do not specifically focus on workplace adversity [6, 7].

A recent meta‐analysis found that globally approximately one in four critical care nurses intend resigning owing to adverse work environments [8]. Each workplace needs to examine the root causes of workplace adversity in its institution because if not addressed, job dissatisfaction and resignations will ensue [9]. Few studies have explored workplace adversity among critical care nurses using a quantitative approach, with most studies focusing on nurses in general and using qualitative methodologies [4, 10, 11].

## 2 Aim

The study sought to determine the organisational factors associated with workplace adversity from the perspective of critical care nurses working in adult, paediatric and neonatal ICU units in public and private hospitals in the Eastern Cape, South Africa. This article is part of a larger research study examining various aspects of workplace adversity, which will be reported on in future articles.

## 3 Design and Methods

A quantitative, cross‐sectional correlational survey design was used to investigate the organisational factors contributing to workplace adversity among critical care nurses in ICUs.

### 3.1 Setting

The research setting was the healthcare system in South Africa, which is divided into two sectors: public and private. The study focused on 11 public and private hospitals in the Eastern Cape, South Africa, with a total of 24 ICUs. Two units were excluded due to their small size and because they largely functioned as high‐care units.

### 3.2 Sampling and Sample Size Calculation

A census sampling method was chosen, with no specific inclusion or exclusion criteria. However, the sample did not include those who were sick or on leave at the time of data collection. Participation was voluntary. It was determined that a minimum sample size of 167 respondents would be adequate for significance and statistical power. However, owing to a good response rate, a larger sample size of 353 respondents participated in the study.

### 3.3 Data Collection Methods

In‐person data collection took place from 26 April 2022 to 4 July 2022, following ethical clearance and permission from various stakeholders. Twelve fieldworkers, trained by the primary researcher, assisted with data collection as the study spanned a wide geographical area. Information regarding the study was provided to prospective respondents by the fieldworkers, who explained each aspect of the self‐report structured questionnaire, the respondent information letter and informed consent. Respondents placed the completed questionnaires and consent forms in separate sealed boxes, which were stored at the nurse's station. The hospitals were grouped and named as Private Hospital Group A, Private Hospital Group B and Public Hospital Group C to uphold anonymity.

### 3.4 Data Collection Tool

The data collection tool was a self‐report structured questionnaire developed using a narrative literature review and guided by the JD‐R Model, as there was no suitable pre‐existing questionnaire addressing the problem of workplace adversity in ICUs. The Cronbach's alpha score for the section on the organisational factors contributing to workplace adversity in the questionnaire was 0.822. This score meant that the items demonstrated good internal consistency, and that the questionnaire reliably captured nurses' perceptions of organisational factors contributing to workplace adversity.

### 3.5 Data Analysis

Data analysis was conducted using Statistica Version 13.5. The descriptive statistics included frequency distributions and percentages. The inferential statistics included one‐sample Wilcoxon signed‐rank tests; inferential ranking; exploratory factor analysis (EFA) and one‐way analysis of variance (ANOVA) tests.

### 3.6 Ethical and Institutional Approval

Nelson Mandela University granted ethical clearance with the approval number H20‐HEA‐NUR‐024 on 3 December 2021. Consequently, permission was sought from the Eastern Cape Department of Health (ECDOH) and private hospital groups with ICUs throughout the Eastern Cape. The ethical principles of the Belmont Report were adhered to throughout the study's duration.

## 4 Results

### 4.1 Demographic Data of Respondents

At the time of data collection, 353 critical care nurses participated in the study. The response rate was 77%. Altogether 28% of the respondents had worked in an ICU for between 10 and 19 years, and the majority (91.6%) of the respondents were female. A third of the respondents (33%) were between the ages of 30 and 39 years. More than half of the respondents (51.2%) reported working in an adult ICU. Most respondents (76.3%) indicated that they worked in the private sector ICUs. More than half of the respondents (52.7%) were working on shifts. A significant number of respondents (59.3%) indicated that they held an additional qualification in critical care nursing, as recognised by the South African Nursing Council (SANC).

### 4.2 Organisational Factors Contributing to Workplace Adversity

The questionnaire items addressing staff shortages, unrealistic expectations, high patient ratios and insufficient knowledge were underpinned by the job demands construct of the Job Demands‐Resources (JD‐R) model. They represent factors that increase nurses' physical and psychological workload [12]. In contrast, items related to limited professional development, poor leadership, inadequate training and lack of evidence‐based protocols reflected a lack of job resources [12], which hindered nurses' ability to manage workplace demands effectively. These constructs provided the theoretical basis for examining the organisational factors contributing to workplace adversity among Critical care nurses. The results derived from these dimensions are presented in Table 1.

**TABLE 1:** Organisational Factors Contributing to Workplace Adversity.

	Strongly disagree		Disagree		Neutral		Agree		Strongly agree		Total	
Insufficient evidence‐based guidelines/resources or protocols to guide me in caring for patients	35	10%	114	33%	61	18%	96	28%	38	11%	344	100%
Shortage of nursing staff	5	1%	9	3%	21	6%	132	38%	180	52%	347	100%
Limited access to professional development	12	3%	71	21%	66	19%	103	30%	94	27%	346	100%
Inappropriate leadership style of direct line managers	23	7%	86	25%	65	19%	95	28%	76	22%	345	100%
Unrealistic expectations in my job description	14	4%	103	30%	81	23%	90	26%	58	17%	346	100%
Insufficient nurse‐to‐critically ill patient ratios	9	3%	42	12%	51	15%	112	32%	131	38%	345	100%
Insufficient knowledge to make clinical decisions regarding patients I care for.	35	10%	134	39%	77	22%	64	19%	35	10%	345	100%
Inadequate formal training at my university or nursing college has not prepared me to manage challenges in my workplace.	72	21%	127	37%	74	22%	41	12%	29	8%	343	100%

Table 1 shows that the majority (*n* = 312, 90%) of the critical care nurses reported that staff shortages contributed to workplace adversity. Most of the respondents (*n* = 243, 70%) strongly agreed or agreed that there were inadequate nurse‐to‐critically ill patient ratios. Regarding professional development, more than half of the respondents (*n* = 197, 57%) strongly agreed or agreed that they had limited access to professional development. Half of the respondents (*n* = 171, 50%) strongly agreed or agreed that the leadership style of their direct line managers was inappropriate. Over a third of the respondents (*n* = 134, 39%) strongly agreed or agreed that there was insufficient evidence‐based guidelines/resources or protocols to guide them in caring for patients.

Table 2 presents the results of the one‐sample Wilcoxon signed‐rank tests conducted to classify the contributing factors of workplace adversity among critical care nurses based on sample median values.

**TABLE 2:** One‐sample Wilcoxon signed‐rank test classification.

Factors	Descriptive statistics				One‐sample Wilcoxon signed‐rank test classification (H_1_: median ≠ 3)				Inferential ranking	
	*n*	Mean	SD	Median	Standardised test statistic	SE	*p*	Category	Rank	Significance grouping
Shortage of nursing staff	334	4.36	0.84	5	−4.362	1735.41	< 0.0005	High	1	1
Insufficient nurse‐to‐critically ill patient ratios	332	3.92	1.12	4	14.357	1722.60	< 0.0005	Middle‐High	2	2
Limited access to professional development	333	3.57	1.19	4	4.96	1744.33	< 0.0005	Middle‐High	3	3
Inappropriate leadership style of direct line managers	332	3.35	1.25	4	1.532	1738.44	0.125	Middle‐High	3	3
Unrealistic expectations in my job description	333	3.21	1.17	3	−0.589	1744.60	0.556	Middle	5	4
Insufficient evidence‐based guidelines, resources or protocols to guide me in caring for patients	332	2.97	1.21	3	9.506	1729.04	< 0.0005	Middle	5	4
Insufficient knowledge to make clinical decisions regarding patients I care for.	332	2.78	1.16	3	−6.962	1732.62	< 0.0005	Middle	7	5
Inadequate formal training at my university or nursing college has not prepared me to manage challenges in my workplace.	330	2.49	1.19	2	−9.878	1718.78	< 0.0005	Low	8	6

Table 2 shows that, according to the median values, the most significant organisational factor that was perceived to contribute to workplace adversity was the shortage of nursing staff, with a median value of 5 (*M* = 4.36). Additional factors with a middle high category were poor nurse‐to‐critically ill patient ratios (*M* = 3.92), limited access to professional development (*M* = 3.57) and inappropriate leadership styles of direct line managers (*M* = 3.35), all with a median value of 4.

The EFA pattern matrix indicated the underlying factor structure extracted with the associated factor loadings. The factor was extracted using the principal axis factoring method with a minimum factor loading of 0.3. The items forming the factors' structure were reviewed and labelled according to the prevalence of perceived organisational factors causing workplace adversity, as illustrated in Table 3.

**TABLE 3:** Exploratory factor analysis.

Items	Factor 1
Insufficient evidence‐based guidelines/resources or protocols to guide me in caring for patients	0.587
Shortage of nursing staff	0.523
Limited access to professional development	0.618
Inappropriate leadership style of direct line managers	0.637
Unrealistic expectations in my job description	0.769
Insufficient nurse‐to‐critically ill patient ratios	0.606
Insufficient knowledge to make clinical decisions regarding the patients I care for.	0.606
Inadequate formal training at my university or nursing college has not prepared me to manage challenges in my workplace.	0.506

Table 3 shows that the primary issues highlighted by the respondents were ‘Unrealistic expectations in my job description’, ‘Inappropriate leadership style of direct line managers’ and ‘Limited access to professional development’. The responses were then split according to hospital groups to determine consistency across the hospital types.

For ‘Unrealistic expectations in my job description’, 46.6% of respondents at Private Hospital Group A indicated strongly agree/agree, followed by Private Hospital Group B, with 54.5% indicating strongly agree/agree and Public Hospital Group C, with 85.9% indicating strongly agree/agree. For ‘Inappropriate leadership style of direct line managers’, Private Hospital Group B had the lowest agreement of 39.4% when selecting strongly agree/agree. This result was followed by Private Hospital Group A, with 46% choosing strongly agree/agree, and Public Hospital Group C, with 68.3% indicating strongly agree/agree. For ‘Limited access to professional development’, Private Hospital Group A had the lowest agreement rate of 35.4% when selecting strongly agree/agree, followed by Public Hospital Group C, with 51.3% selecting strongly agree/agree, and Private Hospital Group B, with 54.5% indicating strongly agree/agree.

Comparisons were conducted using ANOVA, with data analysed according to each demographic variable. Post hoc comparisons were performed using Tukey's Honest Significant Difference (HSD) tests to identify statistically significant differences between groups, as presented in Table 4. The table presents how critical care nurses from different hospitals, with different work experience and ages, had differing perceptions of the prevalence of workplace adversity.

**TABLE 4:** Group comparison of organisational factors contributing to workplace adversity.

Hospital group					
Factor	Hospital Group 1	Hospital Group 2	Mean (SD) (Group 1)	Mean (SD) (Group 2)	*p*
Perceived prevalence of causes of workplace adversity	Private—Hospital Group A	Public—Hospital Group C	3.185 (0.765)	3.642 (0.733)	< 0.001

ICU work experience in years group					
Factor	Years in ICU—Hospital Group 1	Years in ICU—Hospital Group 2	Mean (SD) (Group 1)	Mean (SD) (Group 2)	p
Perceived Prevalence of Causes of Workplace Adversity	1–4 years	10–19 years	3.552 (0.830)	3.214 (0.745)	0.039

Age group					
Factor	Age Group 1	Age Group 2	Mean (SD) (Group 1)	Mean (SD) (Group 2)	*p*
Perceived prevalence of causes of workplace adversity	< 30 years old	60+ years old	3.577 (0.716)	2.967 (0.707)	0.014

Table 4 shows a statistically significant difference (*p* < 0.001) in the perceived prevalence of workplace adversity between respondents employed in Private Hospital Group A and those in Public Hospital Group C. Respondents from Private Hospital Group A reported lower levels of workplace adversity (*M* = 3.185, SD = 0.765). In contrast, those from Public Hospital Group C reported higher levels of perceived adversity (*M* = 3.642, SD = 0.733). This result suggests that critical care nurses' working in the public sector perceived workplace challenges to be more prevalent than their counterparts in the private sector.

Respondents who had worked in an ICU for 10–19 years reported significantly lower levels of perceived workplace adversity (*M* = 3.214, SD = 0.745) compared with those with 1–4 years of ICU experience (*M* = 3.552, SD = 0.830). This result indicated that critical care nurses with less experience perceived workplace challenges as more prevalent than their more experienced counterparts.

Respondents aged 60 years or older perceived the listed workplace challenges to be significantly less prevalent (*M* = 2.967, SD = 0.707) than those younger than 30 years old (*M* = 3.577, SD = 0.716). This result suggested that younger critical care nurses may be more sensitive to, or more affected by, adverse workplace conditions.

## 5 Discussion

The demographics indicate that respondents who had worked in the ICU for 10–19 years did not perceive workplace adversity to be as prevalent or intense as much as the 1–4‐year group. In addition, older respondents perceived workplace adversity as less prevalent or less severe compared with younger respondents, who reported higher levels of perceived adversity. Hence, more experienced, older critical care nurses were less susceptible to adversity, suggesting higher levels of resilience. An integrative literature review found significant positive associations between age and resilience; additionally, also years of experience and resilience [13].

Respondents from both public and private hospitals indicated that staff shortages were a cause of workplace adversity in their respective ICUs. Shortages of critical care nurses are a global problem [14, 15]. Furthermore, inadequate staffing increases workloads, extends shifts and undermines the quality of patient care, causing stress, fatigue and moral distress. Inappropriate staffing levels due to nursing shortages, especially in ICU settings, contribute towards workplace adversity [16]. A US ICU study highlighted the importance of prioritising appropriate staffing practices as these are a major component of a healthy work environment [17].

Limited access to professional development opportunities for critical care nurses contributes to workplace adversity. More than half of the respondents in the current study perceived limited access to their professional development. A study of five European countries indicated that professional development was the fourth highest of 11 factors that would influence critical care nurses to reconsider their intention to leave [18]. Similarly, the Fourth Worldwide Review by the World Federation of Critical Care Nurses (WFCCN) found that insufficient access to quality educational programmes and professional development was the fourth cause of job dissatisfaction [19]. A South African study highlighted that critical care nurses reported challenges to professional development due to inconsistent selection criteria for learning opportunities, subpar training programmes, insufficient budgets and staff shortages that prevent nurses from being released for training [20]. These results indicate a lack of recognition of the need for nurses to have updated skills and knowledge levels through lifelong learning opportunities [21].

An imbalance in critical care nurse‐to‐critically ill patient ratios appears to be a critical driver of workplace adversity. Indeed, three quarters of the respondents supported high nurse‐to‐patient ratios as a cause of workplace adversity in ICUs. Furthermore, the quality of care rendered to critically ill patients is largely determined by nurse‐to‐patient ratios [22]. The ‘Global ICU Needs Assessment’ found that 87 ICUs (72%) across 34 countries had either a 2:1 or ≥ 2:1 patient‐to‐nurse ratio [23]. In analogous surveys in Germany [24] and in South Africa [25], the nurse‐to‐patient ratios in ICUs were spotlighted in unsafe ranges with one nurse to three patients. Chen et al. [26] found a significant relationship between high nurse‐to‐patient ratios and occupational stress, which is a contributor to workplace adversity.

Inappropriate leadership styles adopted by ICU line managers contribute to workplace adversity. When nurse leaders demonstrate toxic leadership, critical care nurses often experience workplace adversity in the form of increased stress and job dissatisfaction [27]. Half of the respondents in the study perceived the leadership style of their direct line managers as inappropriate. This result, supported by a South African survey [28], points to a general concern with leadership practices and highlights the need for improvement in managerial approaches in ICUs. The survey found that corruption and poor leadership in healthcare systems were widespread [29]. When leaders are inadequate, employees disengage and if favouritism is evident, resentment towards line managers occurs [1]. Poor leadership can be detrimental to an organisation as it affects morale, team dynamics, decision‐making or even organisational culture [28].

Unrealistic expectations embedded within critical care nurses' job descriptions are a significant source of workplace adversity. Almost half of the respondents identified unrealistic expectations and felt they were expected to perform duties beyond those outlined in their job descriptions. A Canadian study confirmed that nurses in ICUs are sometimes overwhelmed by their work‐related expectations [10]. The rationale is that they have expectations from themselves, colleagues, management, organisations and regulatory bodies [10]. A qualitative study undertaken in Australia found that when critical care nurses felt their role expectations were not met, it undermined their capacity for care and compassion for their patients [30] and work–life balance was skewed [31].

The absence of sufficient evidence‐based guidelines and resources for caring for critically ill patients contributes significantly to workplace adversity. More than a quarter of the respondents indicated that workplace adversity was caused by the lack of sufficient evidence‐based guidelines or resources to guide them in caring for critically ill patients. Not having reliable, science‐based instructions meant that critical care nurses were unable to practise evidence‐based care, leading to poor patient outcomes. The Fourth Worldwide Review on critical care nursing organisations found that insufficient formal practice guidelines were ranked as the second most significant cause of workplace adversity, limiting nurses' ability to care for critically ill patients [19]. Hence, the limited availability of relevant guidelines within healthcare institutions is one of the main barriers to evidence‐based practice (EBP) [32]. A Palestinian cross‐sectional study of ICUs reported that nurses lacked protocols and EBP guidelines, leading to frustration [33].

In this study, critical care nurses identified several job demands contributing to workplace adversity, including staff shortages, inadequate nurse‐to‐patient ratios, and unrealistic expectations in job descriptions, all of which increase physical, cognitive and emotional workload. Conversely, nurses reported a lack of key job resources, such as limited access to professional development, inappropriate leadership styles and insufficient evidence‐based guidelines. A lack of such resources reduces their capacity to manage these demands effectively. Collectively, these organisational factors create an imbalance between demands and resources, thus heightening workplace adversity in high‐acuity units.

## 6 Study Limitations

Several limitations of this study should be acknowledged. The research was conducted exclusively in the Eastern Cape Province, which may limit the generalisability of the findings to other regions of South Africa. Although data were collected from ICUs in both sectors, 84% of the responses came from private hospitals. This imbalance likely reflects the greater number and size of private healthcare facilities in the province and may have introduced a degree of sectoral bias into the results.

## 7 Recommendations for Practice

Direct line managers and senior management need to constantly identify and address organisational factors that cause workplace adversity in healthcare institutions. They should aim to ensure the availability of clear, evidence‐based guidelines, enhance recruitment and retention strategies to mitigate staff shortages, and ensure that access to professional development opportunities is based on fairness and transparency through policies and procedures. There is also a need to monitor and promote transformational leadership styles to reduce unsuitable leadership. In addition, institutions should implement measurable and realistic job descriptions that include key performance areas (KPAs). Consistent use of nursing workload scoring systems such as the NASA‐TLX can mitigate imbalanced nurse to patient ratios.

## 8 Recommendations for Further Research

The more subtle aspects of workplace adversity, as they relate to the different organisational factors highlighted in this study, could be explored qualitatively through interviews or focus groups involving ICU critical care nurses and especially managers. Intervention research could be used to identify ways to address those organisational factors causing workplace adversity both from a job demands and a job resources perspective.

## 9 Conclusion

The study sought to determine the organisational factors associated with workplace adversity from the perspective of critical care nurses working in adult, paediatric and neonatal ICUs in public and private hospitals. Understanding and addressing these organisational factors should be prioritised to enhance the retention and well‐being of critical care nurses, who are the backbone of any ICU. If these challenges are not resolved, they may destabilise ICU environments when the organisational factors causing workplace adversity become more prevalent. The organisational factors that are evident in ICU settings include increased workloads due to a shortage of trained and qualified critical care nurses, unsuitable leadership, limited opportunities for professional growth, imbalanced nurse–patient ratios and insufficient evidence‐based guidelines. Strategies are needed to equip nurse managers to prepare and support critical care nurses to provide quality care despite challenging organisational factors that contribute towards workplace adversity.

## Funding

The authors thank the National Research Foundation (NRF), South Africa for its financial support (BAAP210416595442).

## Ethics Statement

Nelson Mandela University granted ethical clearance with the approval number (H20‐HEA‐NUR‐024). The approval date was 3 December 2021.

## Consent

The authors have nothing to report.

## Conflicts of Interest

The authors declare no conflicts of interest.

## Data Availability

The data that support the findings of this study are available from the corresponding author upon reasonable request.
